# Comparison of Intraoperative Blood Loss in Patients With Benign Prostatic Hyperplasia Undergoing Bipolar Transurethral Resection of the Prostate With and Without Preoperative Oral Finasteride

**DOI:** 10.7759/cureus.90920

**Published:** 2025-08-25

**Authors:** Asad Abullah, Muhammad Sanan, Zabish Mehmood, Usama Ahmad, Hamna Fayyaz, Alishba Iftikhar, Zeeshan Shafqat, Munazza Sadaf

**Affiliations:** 1 Urology, Sir Ganga Ram Teaching Hospital, Lahore, PAK; 2 Urology, Bahawal Victoria Hospital, Quaid-e-Azam Medical College, Bahawalpur, PAK; 3 Urology, Fatima Jinnah Medical University, Lahore, PAK; 4 Surgery, Central Park Medical College, Lahore, PAK; 5 Medicine, Shalamar Hospital, Lahore, PAK; 6 Surgery, Hameed Latif Hospital, Lahore, PAK

**Keywords:** benign prostatic hyperplasia, blood loss, patients, preventive, trans urethral resection of prostate (turp)

## Abstract

Background

Benign prostatic hyperplasia (BPH) is a common urological condition in aging males. Transurethral resection of the prostate (TURP) is a standard modality used for the surgical management of patients with BPH.

Objectives

The main objective of this study is to compare the blood loss among BPH patients undergoing bipolar TURP, with and without preoperative finasteride.

Methodology

A comparative observational study was conducted in the Department of Urology, Sir Ganga Ram Teaching Hospital, Lahore, Pakistan, from March 2023 to August 2023. A total of 60 BPH patients were distributed into two equally sized groups. The patients in Group A were given finasteride (5.0 mg once daily) for two weeks before undergoing bipolar TURP surgery, whereas the patients in Group B underwent bipolar TURP surgery without finasteride. Intraoperative blood loss was calculated in terms of a decrease in hemoglobin (Hb) level, defined as the difference between preoperative Hb and postoperative Hb, measured 24 hours after TURP.

Results

Baseline variables, including mean age (64.4 ± 4.2 vs. 62.9 ± 5.2 years; p-value = 0.528), mean prostate volume (75.64 ± 12.05 vs. 69.75 ± 12.91 grams; p-value = 0.054), median BMI 23.0 (22.0-24.0) vs. 23.0 (21.0-24.0) kg/m²; p-value = 0.341, and median preoperative Hb level 14.0 (14.0-16.0) vs. 14.0 (13.0-15.0) g/dL; p-value = 0.132, were similar between the two groups. Median postoperative Hb level 14.0 (13.0-15.0) vs. 13.0 (11.0-14.0) g/dL; p-value = 0.005 was significantly different between groups. Median blood loss 0.4 (0.3-0.7) g/dL in the finasteride group was markedly lower than 1.3 (0.9-1.8) g/dL in the control group (p-value < 0.001). The need for blood transfusion, 3.6% in the finasteride group and 13.3% in the control group, was not significantly different (p-value = 0.611). Median hospital stay, 26.0 (24.0-28.0) hours in the finasteride group, was markedly shorter than 28.0 (26.0-30.0) hours in the control group (p-value = 0.011).

Conclusion

The study concluded that preoperative treatment with finasteride for 14 days effectively decreased intraoperative blood loss in patients with BPH who underwent bipolar TURP in our hospital. Therefore, preoperative finasteride can be used to reduce blood loss and associated complications in BPH cases scheduled for bipolar TURP.

## Introduction

Benign prostatic hyperplasia (BPH) is one of the most common urological conditions affecting aging men, characterized by nonmalignant enlargement of the prostate gland due to hyperplasia of both stromal and epithelial components. This condition can lead to lower urinary tract symptoms (LUTS), such as frequency, urgency, nocturia, hesitancy, and incomplete bladder emptying, which significantly impact quality of life [1]. BPH is defined as prostate gland enlargement accompanied by symptoms related to the urinary system and not indicating any cancer. The prevalence of BPH is continuously increasing because of a rise in adaptable metabolic risk factors, like obesity [2].

Patients with BPH who fail to respond to medical therapy as the first line of treatment undergo a surgical intervention. Hemorrhage is the most frequent and critical complication encountered during prostate surgery. In some cases, the bleeding may become severe enough to increase morbidity and even cause mortality. Persistent bleeding often necessitates blood transfusion and can result in clot retention during the perioperative phase [3].

The enlarged prostate causes problems in urination, as its progressive development obstructs the urethra and subsequently results in the obstruction of urine passage. This obstruction can cause urinary tract infections (UTIs) or renal dysfunction [4]. Most of the pathophysiology of BPH is unknown. The traditional view suggests that dihydrotestosterone (DHT), rather than testosterone itself, is the key driver in the development of BPH, as the early stages of prostate growth are strongly dependent on androgenic stimulation mediated by DHT [5].

The size expansion of the prostate gland can result in bladder outlet obstruction (BOO) that can develop LUTS in two different ways: (1) Static component, or thickening of the prostate gland, physically constricts the urethra; (2) dynamic component, or the influence of increased smooth muscle tone [6]. The complications of BPH are UTIs, bladder stones, and persistent kidney issues [7].

The gold standard surgical technique for treating LUTS and BPH is still transurethral resection of the prostate (TURP). Through the use of a resectoscope introduced via the urethra, extra prostate tissue is removed during this minimally invasive surgery, easing blockage and enhancing urine flow [8]. This surgical treatment technique is efficient and cost-effective, with a low complication rate. But hemorrhage, or blood loss, is the most prevalent and serious complication of TURP [9]. The complication of blood loss can be linked with changing the Foley catheter, manual irrigation, and blood transfusion, and can lead to prolonged hospital stay, which increases the hospital stay cost [10].

Consequently, there is a growing interest in identifying strategies to minimize perioperative bleeding and optimize surgical outcomes in BPH patients undergoing TURP. The use of preoperative oral finasteride as an adjunctive therapy to reduce blood loss during TURP procedures is an emerging strategy. Finasteride is a selective inhibitor of 5-alpha reductase, the enzyme responsible for converting testosterone to DHT. By inhibiting DHT production, finasteride effectively reduces prostate volume and vascularity, potentially mitigating intraoperative bleeding [7]. Finasteride and dutasteride, two 5-alpha-reductase inhibitors, are useful drugs for treating androgenic alopecia and BPH [11].

Objective

The objective of this study was to compare the efficacy of misoprostol versus tranexamic acid in reducing intraoperative blood loss during myomectomy, and to evaluate their impact on secondary outcomes, including transfusion requirements, operative time, postoperative recovery, and hospital stay.

## Materials and methods

This comparative observational study was conducted at the Department of Urology, Fatima Jinnah Medical College/Sir Ganga Ram Teaching Hospital, Lahore, Pakistan, from March 2023 to August 2023 (approval no. 158/ERC/FJMU). Written informed consent was obtained from all participants. A total of 60 male patients diagnosed with BPH were enrolled. The inclusion criteria were: (1) age between 55 to 70 years, (2) prostate size between 40 and 100 grams, as measured by ultrasonography (USG KUB) conducted by a consultant radiologist, and (3) BPH associated with complications such as refractory urinary retention, bladder stones, renal insufficiency, hematuria, or recurrent UTIs. Patients were excluded if they had: (1) any known bleeding disorder, (2) were on anticoagulant therapy, (3) had uncontrolled diabetes mellitus, hypertension, or ischemic heart disease, or (4) a confirmed diagnosis of prostate carcinoma.

Data collection

Eligible patients were divided into two equal groups (Group A and Group B; n = 30 each). Patients in Group A received oral finasteride 5 mg once daily for 14 days before undergoing bipolar TURP, whereas Group B underwent bipolar TURP without any preoperative finasteride. All patients underwent standardized bipolar TURP under spinal anesthesia, using a 26 French resectoscope and a bipolar generator. After transurethral insertion of the resectoscope, the bladder was thoroughly visualized, with attention to anatomical landmarks, including the ureteric orifices and verumontanum. A resection channel was created at the 5 and 7 o’clock positions, followed by tissue resection extending to the prostatic capsule between 3 and 9 o’clock. Prostatic chips were evacuated using a Toomey syringe. At the end of the procedure, a 24 French three-way Foley catheter was inserted, and continuous bladder irrigation was initiated. Patients were discharged the following morning with the catheter in situ and were reviewed in the Outpatient Department on day 2 for catheter removal. Postoperative medications included analgesics, antibiotics, and stool softeners. Patients experiencing intraoperative complications, such as TUR syndrome, hemodynamic instability, or adverse anesthetic events (e.g., hypotension, spinal headache, and priapism), were excluded from final analysis, though managed as per institutional protocols. The primary outcome was intraoperative blood loss, assessed by calculating the difference between preoperative hemoglobin (Hb) levels and Hb levels measured 24 hours postoperatively. In patients receiving blood transfusions, the effect of transfusion was adjusted using the approximation that one unit of transfused blood increases Hb by 1.0 g/dL. Secondary outcomes included the need for transfusion (Hb < 8.0 g/dL), number of transfused units, and clinical signs of blood loss (hypotension < 90/60 mmHg, heart rate > 100 bpm, dizziness, shortness of breath, nausea, vomiting, and visible hematuria with clots).

Study limitations in methodology

This study only evaluated early postoperative outcomes within 24 to 48 hours, so it did not include delayed complications. Performance and detection bias may have been introduced because neither patients nor clinicians were informed about the intervention. Patient self-report was used to determine compliance with finasteride therapy; there were no pill counts or compliance checks, so there was uncertainty regarding medication consistency.

Data analysis

Statistical analysis was performed using IBM SPSS Statistics for Windows, Version 26 (Released 2019; IBM Corp., Armonk, NY, USA). Continuous variables, including age, prostate volume, operative time, Hb levels, and estimated blood loss, were expressed as mean ± standard deviation (SD). Independent sample t-tests were used to compare normally distributed variables between the two groups. Post-stratification comparisons for age and prostate volume were also performed using t-tests. A p-value ≤ 0.05 was considered statistically significant.

## Results

Both groups had a similar median age of 65.0 years and BMI of 23.0 kg/m², with no significant difference (p = 0.528 and 0.341, respectively). Preoperative Hb levels were also equal (14.0 g/dL in both groups, p = 0.132). However, postoperative Hb was better preserved in the finasteride group (14.0 vs. 13.0 g/dL; p = 0.005), resulting in significantly lower blood loss (0.4 vs. 1.3 g/dL drop; p < 0.001). The median hospital stay was also shorter in the finasteride group (26 vs. 28 hours; p = 0.011), suggesting a beneficial perioperative effect of finasteride (Table 1).

**Table 1:** Comparison of Median Age, BMI, Hemoglobin Levels, Blood Loss, and Hospital Stay Between Groups The Mann-Whitney U test was used for non-parametric comparison due to skewed distributions. Significant differences were noted in postoperative hemoglobin levels, blood loss, and hospital stay duration (p < 0.05), suggesting a hemostatic and recovery benefit of preoperative finasteride administration.

Variable	Group A (Finasteride)	Group B (Without Finasteride)	Mann-Whitney U	p-value	95% CI for Difference
Age (years)	65.0	65.0	707.5	0.528	-2.1 to 3.8
Body Mass Index (kg/m²)	23.0	23.0	386.5	0.341	-0.6 to 1.5
Preoperative Hemoglobin (g/dL)	14.0	14.0	350.0	0.132	-0.2 to 0.7
Postoperative Hemoglobin (g/dL)	14.0	13.0	262.0	0.005	0.3 to 1.1
Blood Loss (g/dL drop)	0.4	1.3	155.0	<0.001	-1.1 to -0.5
Hospital Stay (hours)	26.0	28.0	283.0	0.011	-2.8 to -0.4

The mean prostate volume was slightly higher in the finasteride group, at 75.67 g, compared to 69.30 g in the non-finasteride group. This difference approached, but did not reach, statistical significance (t = 1.969, p = 0.054) (Table 2).

**Table 2:** Comparison of Mean Prostate Volume Between Groups An independent t-test was used to assess for statistically significant differences assuming normal distribution of the continuous variable.

Variable	Group A (Finasteride)	Group B (Without Finasteride)	t-value	p-value
Prostate Volume (g)	75.67 ± 8.3	69.30 ± 9.1	1.969	0.054

Only one patient (3.6%) in the finasteride group required a blood transfusion, compared to four patients (13.3%) in the group without finasteride. Although the difference favored finasteride use, the p-value of 0.611 indicates it was not statistically significant. However, the trend aligns with the significantly lower postoperative Hb drop and reduced blood loss in the finasteride group, supporting its role in minimizing intraoperative bleeding risk (Table 3).

**Table 3:** Blood Transfusion Requirement Among Study Participants Chi-square test was used to compare categorical variables.

Group	Transfusion	Number of Cases (n)	Percentage (%)	χ² (Chi-square)	p-value
Finasteride	Yes	1	3.60%	0.26	0.611
	No	29	96.40%		
Without Finasteride	Yes	4	13.30%		
	No	26	86.60%		

## Discussion

This comparative observational study aimed to evaluate the efficacy of preoperative oral finasteride in reducing perioperative blood loss among patients undergoing bipolar TURP for BPH. The findings of this study suggest that preoperative administration of finasteride may reduce blood loss during bipolar TURP, as indicated by the lower median drop in Hb levels observed in the finasteride group, compared to the control group. The most notable result was the statistically significant difference in postoperative Hb levels and intraoperative blood loss between the two groups. Patients in the finasteride group had a median Hb drop of only 0.4 g/dL, whereas those in the non-finasteride group experienced a significantly larger drop of 1.3 g/dL (p < 0.001). This reinforces the hypothesis that finasteride reduces prostatic vascularity by inhibiting DHT-induced angiogenesis, thereby contributing to a more hemostatic surgical field [12].

Furthermore, the need for blood transfusion, while not statistically significant, was clinically relevant. Only one patient (3.6%) in the finasteride group required a transfusion, compared to four patients (13.3%) in the control group. Although the p-value was not significant (p = 0.611), the reduced transfusion requirement aligns with previous research findings and underscores the clinical utility of finasteride in high-risk surgical patients [13]. Hospital stay duration was also significantly shorter in the finasteride group (median: 26 hours vs. 28 hours; p = 0.011), likely due to improved hemostasis, reduced postoperative complications, and faster recovery. This finding adds to the economic and logistical benefits of preoperative finasteride administration in surgical planning and patient throughput.

The mean prostate volume was higher in the finasteride group (75.67 g vs. 69.30 g), but the difference was not statistically significant (p = 0.054). This near-significance suggests that finasteride might be even more effective in larger prostates, though further studies with larger sample sizes are required to explore this effect more definitively [14]. In regression analysis, the decrease in Hb level remained the only significant predictor of finasteride use after adjusting for confounding variables (AOR: 0.11; p = 0.002), suggesting a robust and independent association between finasteride use and reduced intraoperative blood loss.

Hospital stay duration was also significantly shorter in the finasteride group (median: 26 hours vs. 28 hours; p = 0.011), which may reflect improved hemostasis, fewer postoperative complications, and faster recovery. However, this outcome could also have been influenced by potential confounders, such as surgeon-related variability in operative technique, differences in perioperative care, or unmeasured comorbidities that might affect recovery time [15].

These findings are consistent with previous research, which has reported similar reductions in blood loss with 5-alpha reductase inhibitors. Previous research has shown that finasteride decreases microvessel density within the prostate and attenuates vascular endothelial growth factor (VEGF) expression, thereby improving surgical visibility and reducing the need for electrocautery [16]. However, some studies have yielded inconclusive or conflicting results, likely due to variations in finasteride dosing duration, prostate size, and surgical techniques (monopolar vs. bipolar TURP) [17].

Various limitations are present in this study. First, statistical power in identifying differences in less common outcomes, like transfusion requirements, was perhaps restricted by the relatively small sample used. Second, the study is single-center, which might limit the projected findings due to regional variations in institutional practices and surgical skills. Third, blinding was not employed, leaving room for observer or performance bias in perioperative care and post-surgery management. Also, to test the proposed mechanism of action of finasteride, examinations of VEGF expression or microvessel density - because objective microvessel evaluations were not conducted - were not performed, making it problematic to affirm whether finasteride can serve as a hemostatic agent. Larger cohorts and mechanistic analyses in future multicentric, blinded trials are justified to confirm these results and reinforce causal inference.

## Conclusions

It is concluded that short-term preoperative finasteride use appears effective in reducing intraoperative blood loss in patients undergoing bipolar TURP. The randomized controlled design lends support to this association; however, the absence of blinding, small sample size, lack of adherence confirmation, and single-center setting warrant caution in interpreting these findings. Further large-scale, multicenter trials, with rigorous adherence monitoring, are needed to validate these results and strengthen causal inference.
